# Pain science and practice as a ‘threshold concept’ within undergraduate and pre-registration physiotherapy education: a jewel of the curriculum?

**DOI:** 10.1186/s12909-023-04733-z

**Published:** 2023-10-06

**Authors:** Keith M. Smart

**Affiliations:** 1 UCD School of Public Health, Physiotherapy and Sport Science, University College Dublin, Dublin, Ireland; 2 UCD Centre for Translational Pain Research, Dublin, Ireland; 3 Physiotherapy Department, St. Vincent’s University Hospital, Dublin, Ireland

**Keywords:** Pain, Threshold concept, Physiotherapy, Education

## Abstract

**Background:**

Threshold concepts describe learning experiences that transform our understanding of a concept. Threshold concepts are variously: troublesome, transformative, irreversible, integrative and bounded.

**Purpose:**

The aim of this narrative review is to consider the case for characterising pain science and practice as a threshold concept within undergraduate and pre-registration physiotherapy education.

**Summary:**

This article considers the underlying tenets of threshold concepts as they relate to teaching and learning and the relative merits and limitations of characterising pain science and practice as a threshold concept within undergraduate and pre-registration physiotherapy education from both pedagogical and epidemiological perspectives. By evaluating pain, as it relates to physiotherapy education and practice, according to the five defining characteristics of a threshold concept then presenting data related to the epidemiology and impact of pain, the worthiness of characterising pain science and practice as a threshold concept will be discussed and further debate invited.

## Background

Threshold concepts have been defined as *“concepts that bind a subject together, being fundamental to ways of thinking and practising in that discipline”* [1]. They essentially describe learning experiences that transform our understanding of a concept. Understanding threshold concepts, it has been suggested, are necessary for mastery of a subject [2]. Threshold concepts are thought to aid teaching and learning by attempting to make a distinction between the core learning outcomes of a programme and those learning outcomes that give rise to a new way of looking at a subject [3]. They have also been colloquially termed ‘jewels of the curriculum’ [4].

From a pedagogical perspective, to be considered as such, a threshold concept should variously possess one or more of the five following characteristics and be [3]:

1. Troublesome: involving knowledge that is counter-intuitive, incoherent or challenging;

2. Transformative: involving knowledge that, once understood, causes a significant shift in the understanding of a concept;

3. Irreversible: involving knowledge that is unlikely to be forgotten or unlearned;

4. Integrative: involving knowledge that reveals and demonstrates the interrelatedness of concepts.

5. Bounded: involving knowledge that has frontiers bordering with thresholds into new conceptual areas, or knowledge that defines a particular conceptual field, creating a specific space of expertise within each discipline [5].

In addition to the pedagogical requirements of threshold concepts, the epidemiological data concerning the prevalence, impact and cost of pain/chronic pain from a societal perspective may usefully inform the discussion as to the relative merits of characterising pain as a threshold concept within undergraduate and pre-registration physiotherapy education. ‘Pain’, in this context, refers to the sensory and emotional experience of clinically encountered ‘pain’ [6] as experienced by people attending for healthcare.

### Purpose

To the best of the author’s knowledge, the merits of characterising pain science and practice as a threshold concept in physiotherapy education has not been previously discussed. The aim of this debate article is to consider the case for characterising pain science and practice as a threshold concept within undergraduate and pre-registration physiotherapy education. Pedagogical and epidemiological perspectives will be considered.

## Pain science and practice as a threshold concept: the pedagogical perspective

Pedagogically, the appropriateness and worthiness of designating pain as a threshold concept can be determined by the extent to which it satisfies the five defining characteristics of a threshold concept.

### The troublesome nature of pain science and practice

The complex and troublesome nature of the science and management of pain has been succinctly characterised as ‘The Puzzle of Pain’ [7].

Pain science and practice could be considered troublesome to study and understand from a number of perspectives. Firstly, underlying its complexity is the multidimensionality of the pain experience, with its perceptual (i.e. sensory-discriminative, affective-motivational, and cognitive-evaluative), as well as ontological (i.e. to do with its nature), epistemological (i.e. to do with ways of knowing), linguistic (i.e. to do with language as a conduit to human interaction) and existential (to do with meaning and purpose) dimensions [8–11]. These dimensions can be incredibly challenging to understand and reconcile educationally and clinically.

Secondly, the perception and experience of pain is inherently individual and subjective, and conflicts can exist between patients self-reported experiences and clinicians’ understanding of the clinical significance of pain [12]. In short, it is not possible for a clinician to ‘know’ another person’s pain.

Thirdly, clinical presentations of pain can be complex and troublesome to understand and treat. For example, interpretations of pain based on the biomedical model, which assumes that all pain is caused by injury or pathology, that the severity of pain is proportionate to the underlying cause and that treating the injury/pathology should be accompanied by relief of pain, do not explain either the complexity or variability inherent within many clinical presentations of pain where [10, 13]:

i) pain is reported in the absence of any clearly identifiable pathology;

ii) pain is reported to persist after healing or resolution of pathology;

iii) pain is absent despite evidence of injury or pathology;

iv) the severity of self-reported pain appears, from the clinicians perspective, to be at odds with the severity of injury or pathology;

v) patients’ reports of pain severity in response to similar severities of injuries differs greatly;

vi) relationships between pain, impairment and disability are unpredictable and incongruous;

vii) pain severity is discordant with medical investigations (e.g., radiological imaging);

viii) where patients’ pain responses to identical interventions for the same injury or pathology are highly variable;

ix) paradoxically, pain is absent despite evidence of injury/pathology.

Encountering such variations in clinical presentations of pain could be seen as troublesome for students (and clinicians) to understand [13].

Fourthly, both the International Association for the Study of Pain (IASP) and the European Pain Federation (EFIC) recommend that physiotherapists should assess pain from a biopsychosocial perspective (i.e. the biological, psychological and social dimensions of the pain experience) [14, 15]. However, evidence suggests that some physiotherapists have some difficulty in applying the biopsychosocial model of pain in clinical practice in part because of its perceived complexity [16–19]. Others have sought to demonstrate the complex and challenging nature of pain by deconstructing the biopsychosocial and other contemporary models of pain and inviting the acceptance of pain as potentially insoluble [10].

Together, these assertions highlight the troublesome nature of clinically encountered pain as a focus for teaching and learning.

### Knowledge and understanding of pain science and practice is transformative

Pain science and practice has evolved significantly over the last few decades and these developments, it has been suggested, have had a significant impact on physiotherapy theory and practice [20]. Parker and Madden [20] argue that developments in pain sciences have shifted physiotherapists’ understanding of pain and approaches towards its assessment and treatment, expanded physiotherapy practice, elevated the status of the physiotherapy profession and led to the development of interdisciplinary teams in which physiotherapists play a prominent role.

Theoretically, learning about contemporary approaches to understanding, assessing and managing pain, such as the biopsychosocial model of pain and pain mechanisms-based approaches may be transformative for undergraduate/pre-registration physiotherapy students as they encounter clinical presentations of pain. Such approaches may provide more sophisticated explanations for pain, and its assessment and treatment, beyond those offered by the more reductionist biomedical model [13].

A recent systematic review and meta-analysis found evidence of improved student/qualified health care professionals (including physiotherapists) pain-related knowledge and attitudes and an increased likelihood of clinical behaviour more in keeping with evidence-based practice in response to biopsychosocial focused education strategies [21]. And a recent qualitative evidence synthesis found evidence that biopsychosocial-oriented training for qualified physiotherapists changed the way some considered musculoskeletal pain and its management, changed parts of their practice to a more biopsychosocial framework, improved their confidence in managing musculoskeletal pain and made their work more rewarding [17].

An expanding body of evidence shows that a brief pain neuroscience education session can improve undergraduate physiotherapy students pain knowledge in the short term and positively shift their pain attitudes towards people in pain in the short and medium term [22–26].

Collectively, these findings suggest that modern pain education is capable of at least changing, if not transforming, physiotherapists pain-related clinical knowledge and practice.

### Knowledge and understanding of pain science and practice is irreversible

While there is some evidence to show that appropriate pain education can improve pain-related knowledge and attitudes associated with the biopsychosocial aspects and neurophysiology of pain among undergraduate physiotherapy students (as described above) in the short- and medium-term, the extent to which such knowledge and attitudes are ‘irreversible’, that is unlikely to be forgotten or unlearned, is not known. Previous studies investigating pain neurophysiology [22–26] or biopsychosocial-based pain education [21] rarely, if ever, employ long term follow-up.

Evidence from qualified physiotherapists shows that for some, their clinical reasoning (and practice) remains, to some extent, grounded within the biomedical model of pain despite education in and knowledge and experience of biopsychosocial approaches. The reasons for this are not fully known and could reflect either difficulties in applying such knowledge, as has previously been shown [16–19] or the loss or replacement of knowledge. Further research could explore this.

Currently, the extent to which physiotherapists knowledge and understanding is irreversible is not known.

### Pain science and practice is integrative

Clinically encountered pain is ubiquitous across healthcare-related settings, disciplines, specialisms and conditions. Therefore, knowledge and understanding associated with pain science and clinical practice has the potential to reveal and demonstrate to physiotherapy students its interrelatedness with a myriad of other health-related constructs, concepts and body systems [27]. The literature is replete with evidence of how the sensory and emotional experience of pain is interconnected with emotions [28], cognitions [29], disability [30, 31], the social environment [32], risk factors [33], as well as the nervous [34], immune [35], endocrine [36], stress [37, 38] and cardiovascular systems [39].

A knowledge and understanding of pain developed through integrative pain education could help physiotherapy students begin to appreciate the interrelatedness of pain and develop meaningful connections between pain and broader health-related concepts. Future research could explore this.

### Pain as a bounded concept

At the same time as being highly integrative pain science and practice is internationally recognised as a scientific and clinical discipline as represented by the International Association for the Study of Pain (see https://www.iasp-pain.org/advocacy/iasp-statements/desirable-characteristics-of-national-pain-strategies/).

Furthermore, the assessment and management of pain are bounded by core outcome sets [40], clinical guidelines [41], standards of care [42], and contributes to an international disease classification system that considers chronic pain to be a condition in its own right and not solely a symptom of diseases and injuries [43].

Collectively, these findings confirm pain as a particular clinical specialty that has created a specific space of expertise within and between medical and scientific professions and disciplines.

## Pain science and practice as a threshold concept: the epidemiological perspective

Understanding pain and its clinical presentations is vital given its prevalence and adverse personal and socioeconomic impact. Approximately 20–30% of the adult populations of Europe and United States of America are affected by chronic (typically ≥ 3 months in duration) pain [44–47]. Pain, and pain-related conditions (e.g., low back and neck pain) are leading causes of disability and disease burden globally [48]. Two pain-related conditions (arthritis and back pain) are included within the top 10 most common conditions for which consultations are sought in primary care globally [49].

Chronic pain can have a profound adverse impact on the daily activities, quality of life and mental health of those who suffer with it, together with wider consequences on home, work, and social life [50, 51]. The economic costs arising from healthcare expenditure, lost work productivity, absenteeism, and early retirement secondary to chronic pain can be enormous to nations, running into billions annually [44].

In light of these findings, chronic pain is becoming increasingly viewed as a public health concern [52–54]. Data demonstrating the extent and impact of pain/chronic pain globally could be used to support the case for characterising pain science and practice as a threshold concept within undergraduate/pre-registration physiotherapy education.

### Pain science and practice education within physiotherapy programmes

Given that (chronic) pain is common and costly and that trainee physiotherapists, regardless of clinical speciality and setting, are frequently confronted with clinical presentations of pain as they undertake clinical placements it is incumbent on physiotherapy educators to ensure that student physiotherapists acquire the necessary knowledge and clinical skills required to understand, assess and manage it optimally.

Evidence suggests that pain education within undergraduate healthcare training programmes, including physiotherapy, has long been insufficient and could be improved in order to meet best practice standards [55–60].

Guidelines for incorporating pain education into undergraduate curricula for healthcare professionals have been published [61, 62] and reforming physiotherapy curricula to support students to develop clinical competencies based on current pain neuroscience has been advocated [63].

Pain curricula to improve pain education within undergraduate/pre-registration physiotherapy programmes and across the professional lifespan have been developed by the IASP [13] and EFIC [14] respectively. The IASP curriculum has subsequently informed the development and revision of undergraduate physiotherapy pain education in the United States [63] and Australia [64].

Recognition of pain science and practice as a threshold concept could provide the impetus to improve pain-focused teaching and learning within undergraduate and pre-registration physiotherapy programmes and encourage others to implement guidelines and revise the nature and extent of pain education and content within their curricula.

### Limitations

This article presents the perspective of one academic and clinical physiotherapist with a special interest in pain science and practice. It is hoped that this article might stimulate debate within the profession, among physiotherapy students, educators, clinicians, researchers, managers and professional regulators, as to the relative merits of characterising pain science and practice as a threshold concept within undergraduate and pre-registration physiotherapy education, and potentially, across the professional lifespan. It may also stimulate wider debate regarding the identification of those threshold concepts upon which physiotherapy education and training could or should be based.

Understanding of threshold concepts continues to evolve and there is currently no agreement on how they should be identified or designated. For example questions such as, ‘How many of the aforementioned five characteristics should a concept possess to be considered a threshold concept?’ or ‘Are some characteristics more important than others?’ remain unanswered [65]. Interpretations of the criteria vary, and additional characteristics associated with threshold concepts, such as being ‘*discursive*’ and ‘*reconstitutive*’, have also been described [65]. As such, methods for the identification and designation of threshold concepts remain ambiguous and somewhat arbitrary [66, 67] although various research methods, such as consensus-building processes, could be employed to facilitate this.

Also, there are no known and accepted ways to ‘measure’ or ‘judge’ the extent to which a concept satisfies the defining criteria of threshold concept, i.e. extent to which a given concept is *troublesome* or *transformative*. Consequently, the identification or designation of threshold concepts remains problematic, although a framework to assist educators to identify and embed threshold concept knowledge into their programmes has recently been described [68].

Furthermore, having knowledge of pain science and practice in no way guarantees that patients’ care and outcomes will be enhanced [69].

## Summary

The relative merits of characterising pain science and practice as a threshold concept within undergraduate and pre-registration physiotherapy education have been considered. Pedagogically it appears that pain science and practice is, to varying degrees, troublesome, transformative, integrative and bounded. The extent to which it is irreversible is not known. Epidemiologically, it could be argued that the prevalence, impact and costs associated with pain/chronic pain from a societal perspective are of sufficient magnitude to support the characterisation of pain science and practice as a threshold concept. In the absence of accepted methodologies with which to identify threshold concepts this paper presents the perspective of a single author. Recognition of pain science and practice as a threshold concept could provide the impetus to improve pain education within undergraduate and pre-registration physiotherapy. Wider debate regarding the relative merits of characterising pain science and practice as a threshold concept would be welcome.

## Data Availability

Not applicable.
